# Cellular and molecular basis of neuropathic pain

**DOI:** 10.1136/rmdopen-2025-006044

**Published:** 2026-03-11

**Authors:** Pao-Sheng Chang, Annina B Schmid, Franziska Denk

**Affiliations:** 1 Nuffield Department of Clinical Neurosciences, University of Oxford, Oxford, UK; 2 Wolfson Sensory, Pain and Regeneration Centre (SPaRC), King’s College London, London, UK

**Keywords:** Pain, Inflammation, Analgesics

## Abstract

Neuropathic pain is a devastating type of pain that significantly reduces the quality of life of affected people. Traditionally considered as mechanistically distinct from pain induced by classical inflammatory states, studies continue to reveal more commonalities than differences, with a whole host of pathological changes in the environment of local peripheral nerves accompanying chronic neuropathic pain conditions. This narrative review provides an overview of the cellular and molecular drivers of neuropathic pain, highlighting some of the seminal publications from past and present. We discuss both neuronal and non-neuronal mechanisms contributing to neuropathic pain (eg, immune and stromal cell dysregulation). Particular attention is given to studies involving human cohorts which, until recently, have been less common in the field, due to the difficulties in accessing relevant tissues, like nervous system samples. The consequences of recent findings for analgesic drug development are also discussed, both in the context of neuropathic and non-neuropathic pain.

**WHAT IS ALREADY KNOWN ON THIS TOPIC**

- Neuropathic pain has traditionally been studied through a neuroscientific lens, focusing on nervous system abnormalities.

**WHAT THIS STUDY ADDS**

- This review summarises our current state of knowledge of the pathophysiological mechanisms of neuropathic pain, which include dysfunction not only at the neuronal level but also extensive changes to local immune, glial and connective tissue cells.

**HOW THIS STUDY MIGHT AFFECT RESEARCH, PRACTICE OR POLICY**

- Our review highlights promising avenues for analgesic drug development.

## Introduction

 Most rheumatic and musculoskeletal conditions have pain as one of their primary symptoms. This pain can be experienced in many forms (box 1), and sometimes individuals describe shooting, burning or tingling sensations, raising the possibility that they are experiencing neuropathic pain. Neuropathic pain is ‘caused by a lesion or disease of the somatosensory nervous system’.^1^ A typical example would be radiating leg pain caused by an irritated nerve root in the lower back. However, neuropathic pain has also been suspected in a proportion of people living with persistent arthritis pain^2^: after surgery in osteoarthritis, where nerves might have been damaged,^3^ but also in the absence of an overt cause of nerve injury. For example, a meta-analysis of people with knee and hip osteoarthritis estimated an overall prevalence of possible neuropathic pain of 23% (95% CI 10% to 39%).^4^

Box 1 Pain is defined as ‘an unpleasant sensory and emotional experience associated with, or resembling that associated with, actual or potential tissue damage’.^1^ As such, pain is a personal and complex experience generated by the brain and influenced not just by sensory input, but also by emotional and motivational input. Chronic pain is commonly described in the context of a bio–psycho–social model, acknowledging that the ultimate pain experience is influenced by a variety of complex factors.^89^

Currently, three terms are used to broadly describe the different mechanisms that can drive chronic pain, defined as follows by the International Association for the Study of Pain (IASP)^1^:

- Nociceptive pain: ‘pain that arises from actual or threatened damage to non-neural tissue and is due to the activation of nociceptors’.
- Neuropathic pain: ‘pain caused by a lesion or disease of the somatosensory nervous system’.
- Nociplastic pain: ‘pain that arises from altered nociception despite no clear evidence of actual or threatened tissue damage causing the activation of peripheral nociceptors or evidence for disease or lesion of the somatosensory system causing the pain’.

It is important to note that these are descriptive terms, rather than diagnostic categories, and they often coexist in the form of mixed pain. For diagnostics, chronic pain is distinguished in the International Classification of Diseases, 11th Revision (ICD-11) according to its likely origin^90^: chronic primary pain, chronic cancer-related pain, chronic postsurgical or posttraumatic pain, chronic neuropathic pain, chronic secondary headache or orofacial pain, chronic secondary visceral pain, chronic secondary musculoskeletal pain, other specified chronic pain and unspecified chronic pain.

A limitation of such reports is that they largely had to rely on patient-reported outcome measures; yet, symptomatically, pain due to nerve damage is very difficult to distinguish from severe, widespread musculoskeletal pain.^2^ Indeed, neuropathic pain is far from easy to conclusively diagnose: current grading^5^ and assessment^6^ criteria, developed mostly in the context of systemic diabetic and/or small fibre neuropathy, require detailed clinical assessments, including sensory signs that indicate nerve damage in a neuroanatomically plausible fashion, and a diagnostic test that confirms a lesion or disease of the somatosensory system. For example, a nerve conduction study can confirm whether large axons are sending electrical signals more slowly than they should, or a skin biopsy might show that nerve endings have degenerated, as would typically be the case in diabetic neuropathy. Questionnaires alone are considered insufficient for clinical diagnosis and are likely to overestimate rates of neuropathic pain, a challenge that is fully acknowledged in the field.^2^,^4^

And yet, it is vital that we strive to overcome those challenges and identify the precise origins of pain in different individuals. This is particularly important in the case of neuropathic pain, against which ‘classical’ painkillers, that is, non-steroidal anti-inflammatories and opioids, are largely ineffective. Indeed, the most recent recommendations for first-line treatments for neuropathic pain, published in 2025 by the Neuropathic Pain Special Interest Group, are tricyclic antidepressants, serotonin and norepinephrine reuptake inhibitors and α2δ-ligands, such as gabapentin and pregabalin.^7^ These are ostensibly ‘old’ compounds, but exciting possibilities are on the horizon for analgesic drug development. This is because recent research into neuropathic pain is starting to uncover novel mechanisms that might allow for more precise targeting of damaged neurons and their local tissue environments. Some of these targeting strategies also have the potential to translate to other, non-neuropathic musculoskeletal conditions.

**Figure 1:**
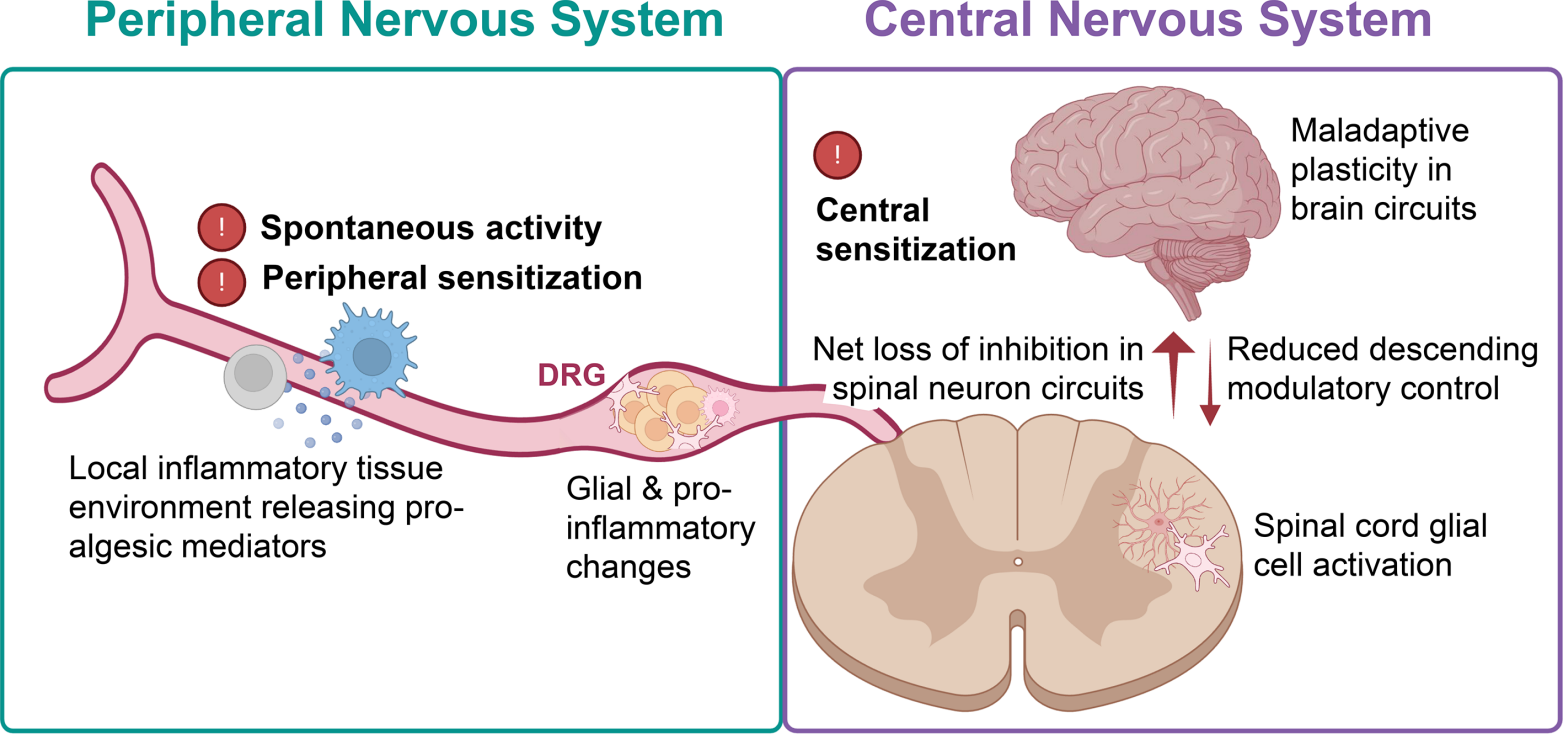
Biological mechanisms underlying neuropathic pain conditions of peripheral origins. Nerve damage or disease in the periphery leads to proalgesic mediator release from local inflammatory tissues, as well as glial and proinflammatory changes in the dorsal root ganglion (DRG), where the cell bodies of sensory neurons are located. Functionally, peripheral neurons become hyperactive, displaying spontaneous activity as well as peripheral sensitisation, that is, a lowering in their response thresholds. Neuropathic pain is also accompanied by sensitisation of central nervous system circuits; these include a net loss of inhibition in the spinal cord, accompanied by glial cell activation, maladaptive plasticity changes in the brain, as well as reduced activity in descending modulatory circuitry. Created in BioRender by P-SC: https://BioRender.com/lf6t2wu.

In the following narrative review, we will summarise the current evidence base for the various cellular and molecular drivers of neuropathic pain (figure 1), highlighting seminal historical literature alongside recent advances.

### Nervous system abnormalities in neuropathic pain

Given that neuropathic pain, by definition, involves a lesion or disease of the somatosensory system, it is only logical that, historically, most research has focused on the neuropathological origins of the condition. In a neuropathic pain state, abnormalities can be observed at all levels of the sensory pathway, including the peripheral neurons, the spinal cord and higher cortical areas.

Peripheral sensory neurons are pseudo-unipolar cells, extending one axon into the body and one into the central nervous system. Their cell bodies are located in ganglia just lateral to the spinal cord or cauda equina, within intervertebral foramina. In healthy individuals, peripheral sensory neurons are largely silent, that is, they do not fire any action potentials. This is in contrast to central nervous system neurons, many of which display tonic electrical activity. In neuropathic pain states, so-called spontaneous activity has been consistently observed in all types of peripheral sensory fibres,^8^ both in animal models and in individuals living with neuropathic pain, where the activity of small unmyelinated sensory fibres can be recorded with a highly specialised technique known as microneurography.^9 10^ Associations have been observed between the level of spontaneous activity recorded in these fibres and the level of neuropathic pain experienced by individuals.^11 12^ The cause of spontaneous sensory neuron activity is still not entirely clear; early seminal papers suggest that it can originate at the site of nerve damage as well as in the cell bodies of the dorsal root ganglia themselves.^13 14^ Molecularly, increased excitability is likely to be related to alterations in ion channel function, for example, mutations in sodium channels can cause abnormal spontaneous firing and lead to rare, painful syndromes, like erythromelalgia.^15^ Equally, of course, it is also possible that some of the abnormal activity is not truly ‘spontaneous’, but rather the result of endogenous mediator release within the neuron’s local tissue environment.

Such mediators have also been linked to the second pathological hallmark displayed by sensory neurons in neuropathic pain, known as peripheral sensitisation. Peripheral sensitisation means that neurons show an increase in their firing rates, either in response to their ‘usual’, preferred stimuli, or in response to novel stimuli that ordinarily would not elicit a response at all.^16^ In people, these neurophysiological properties can only be measured indirectly, assessing for the presence of hyperalgesia (increased pain in response to painful stimulation) and allodynia (pain to normally non-painful stimulation), both of which are present in neuropathic pain states.^17^ On a molecular level, it has been demonstrated that peripheral sensitisation can be induced by soluble, proinflammatory mediators in the neuron microenvironment, such as prostaglandin E2, serotonin, nerve growth factor (NGF), tumour necrosis factor alpha (TNF-α), bradykinin and histamine.^18 19^ Their presence has been reported to cause alterations in neuronal ion channel function and receptor expression. For example, NGF has been demonstrated to increase expression, phosphorylation and trafficking of the transient receptor potential V1 channel.^20^ It remains uncertain whether peripheral sensitisation can persist in the absence of the mediators which induce it, that is, whether the neuron can permanently alter its excitability state through persistent regulation of ion channels. Chronic neuropathic pain is often observed seemingly in the absence of ongoing inflammation, for example, in the case of postherpetic neuralgia, where neuropathic pain persists after a shingles infection. Yet, it is unclear whether this is due to long-term alterations within peripheral sensory neurons or within their local tissue environments or both. Mechanistic explanations are further complicated by the fact that chronic pain conditions, including neuropathic ones, result in significant alterations within central nervous system circuitry, as will be discussed in the following.

Peripheral sensory neurons terminate in the dorsal horn of the spinal cord, amidst a highly complex interneuron network made up of nearly 20 different excitatory and inhibitory cell subtypes.^21^ Information about nociception is then transmitted to the brain via projection neurons in the anatomical tracts of the anterolateral system. Neuropathic pain is accompanied by several changes at the spinal cord level. Most well studied, perhaps, is central sensitisation, described in rodent electrophysiology as the observation that repetitive afferent nociceptive peripheral nerve input leads to increased responsiveness of spinal cord neurons.^22^ In humans, central sensitisation can only be observed indirectly using psychophysical correlates, like temporal summation (also called wind-up ratio) and dynamic mechanical allodynia. Temporal summation is a test with good reliability across clinical cohorts as diverse as rheumatoid arthritis, low back pain and polyneuropathy,^23^ which examines whether pain perception is amplified by repetitive noxious stimuli compared with a single stimulus. Dynamic mechanical allodynia exploits the phenomenon that in pain states, brushing the skin may be perceived as painful. There is good evidence to suggest that dynamic mechanical allodynia is a result of central sensitisation.^24^ While dynamic mechanical allodynia and increased temporal summation are features of neuropathic pain,^25 26^ they are only present in a small subgroup of patients with painful polyneuropathies (~20% dynamic mechanical allodynia, 7% temporal summation), but more common in postherpetic neuralgia (~49% dynamic mechanical allodynia, ~18% temporal summation).^27^ Beyond central sensitisation, neuropathic pain-induced alterations at the spinal cord level have primarily been observed in animal models, where a net loss of inhibition has been described in interneuron circuits, alongside hyperexcitability of projection neurons.^28 29^

Neuropathic pain also induces alterations in higher centres including the brain, where pain is ultimately generated.^30 31^ The brain controls what we experience and can directly modulate spinal cord circuits via a number of descending neuronal pathways, the precise molecular details of which are currently being dissected.^32^,^35^ Animal models of injury-induced neuropathy indicate that there are complex changes to these descending pathways, which may contribute to the maintenance of pain.^28^ Brain imaging suggests that the same may be true in people, for example, pain intensity in diabetic neuropathy is associated with altered connectivity of the ventral periaqueductal grey, one of the main brainstem nuclei involved in descending pain modulation.^36^ Moreover, electrical activity across the brain in general, as measured by electroencephalography, has successfully been used to stratify individuals into those with and without neuropathic pain.^37^

### Non-neuronal cells in neuropathic pain

**Figure 2:**
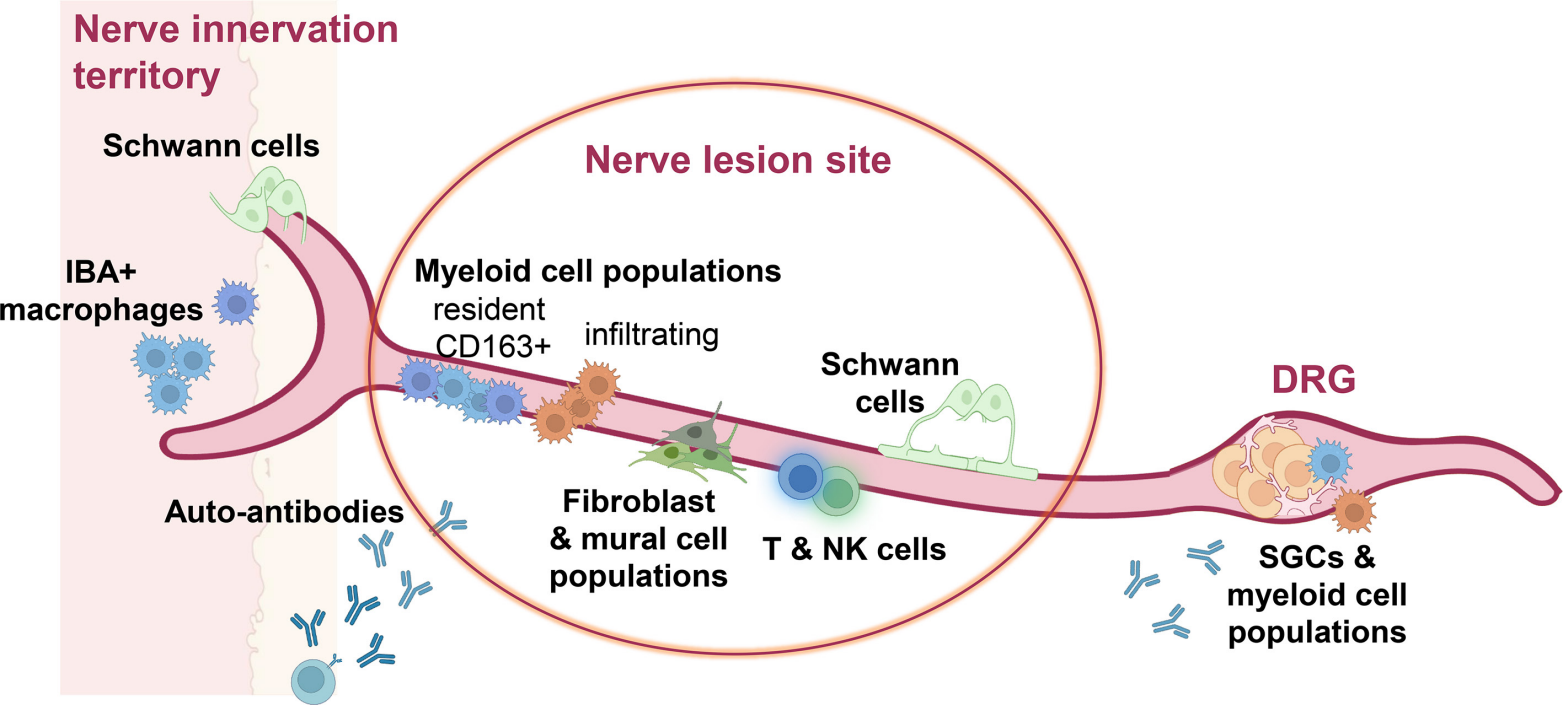
Non-neuronal cell involvement in neuropathic pain in the periphery. Peripheral neuropathic pain conditions are accompanied by significant changes to the non-neuronal cell environment, not only at the site of nerve lesion or disease, but also in the innervation territory of the affected nerves (target tissues), as well as in their cell bodies in the dorsal root ganglia (DRG). Created in BioRender by P-SC: https://BioRender.com/lf6t2wu. IBA1, ionised calcium-binding adapter molecule 1; NK, natural killer; SGCs, satellite glial cells.

Very early on in neuropathic pain research, it was clear that the local neuronal environment can contribute to pathological electrical signalling and pain. For example, Pat Wall, one of the fathers of pain research, highlighted that spontaneous sensory neuron activity and pain can be caused by a neuroma, where damaged peripheral axons are surrounded by inflammatory cells, neovascularisation and fibrosis.^38^ Since then, the field revealed an important role for many non-neuronal cell types in neuropathic pain (figures2  3). We will discuss the resulting evidence in the following, starting with myeloid cells, which are perhaps one of the best-studied cell types in this context.

**Figure 3:**
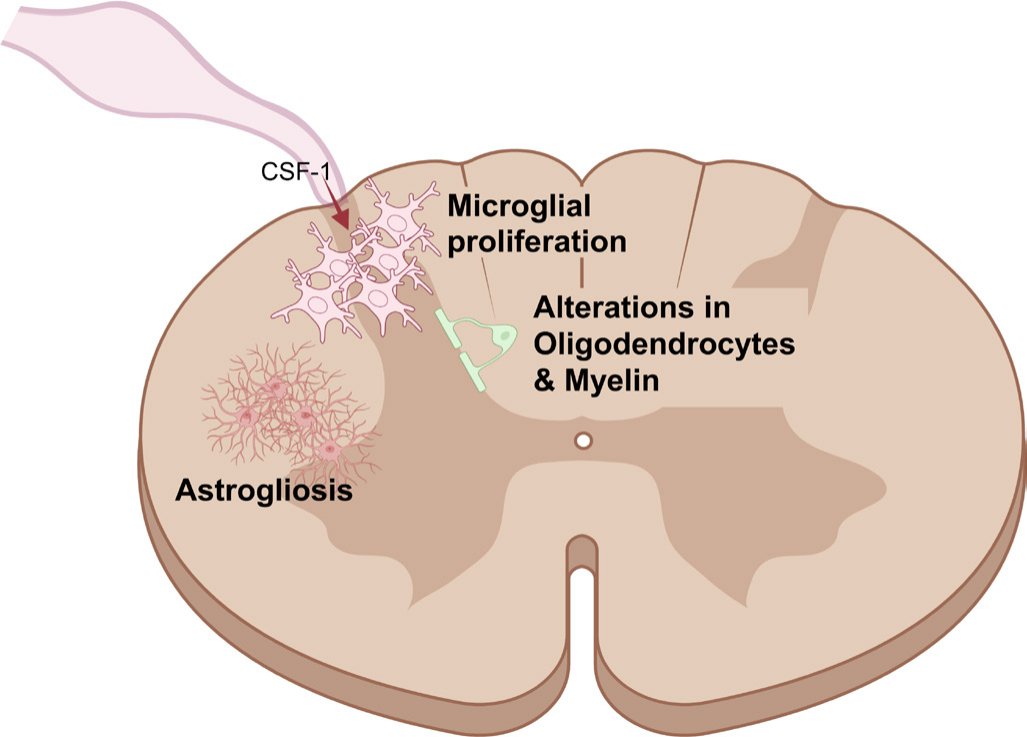
Non-neuronal cell involvement in neuropathic pain in the spinal cord. Damage or disease of peripheral nerves also leads to changes in immune and glial cell function in the spinal cord, including microglial proliferation, astrogliosis and alterations in oligodendrocytes and myelin. However, to date, most of the evidence for these changes derives from animal models. Created in BioRender by P-SC: https://BioRender.com/lf6t2wu. CSF-1, colony-stimulating factor 1.

#### Myeloid cells

Both resident macrophages and infiltrating monocytes have long been linked to painful peripheral nerve injury.^39^ Abnormal myeloid cell numbers are observed both in nerve, at the site of injury, as well as in the dorsal root ganglia, where the neuronal cell bodies are located. In animal models, the changes are most obvious and long-lasting in nerve,^40^ although functionally it has been claimed that myeloid cells in ganglia might be more important than those in nerve when it comes to maintaining neuropathic pain in mice.^41^ Crucially, there have also been a number of studies using human tissues and cohorts, which confirm the presence of abnormal myeloid cell populations beyond rodents. This includes early studies examining sural nerve biopsies from individuals with inflammatory neuropathies, such as Guillain-Barré syndrome^42^ and chronic inflammatory demyelinating polyneuropathy.^43^ Moreover, alterations in macrophage numbers have been observed in the skin of diabetic neuropathy patients, although with opposing results for type I and II diabetes; the former showed a decrease in ionised calcium-binding adapter molecule 1 positive (IBA+) macrophage numbers in samples from those with painful neuropathy,^44^ while the latter showed an increase compared with non-painful neuropathy controls.^45^ Macrophage dysfunction was also highlighted in bulk transcriptomic data of human dorsal root ganglia,^46^ as well as recent single nucleus and spatial transcriptomic data, which implicated a particular macrophage subtype (titled Macro1 by the authors). Macro1 macrophages were found enriched in sural nerves donated by individuals with different types of inflammatory and idiopathic polyneuropathies.^47^ They are likely residents (since they are positive for markers such as CD163) and express the scavenger receptor MARCO. CD163^+^MARCO^+^ macrophages have also been identified as enriched in individuals with a focal neuropathy of the foot and, crucially, *MARCO* mRNA levels were correlated with paroxysmal pain severity.^48^

One fascinating feature of neuropathic pain is that, at least in animal models, peripheral nerve damage appears to recruit microglia, the resident macrophages of the spinal cord.^49 50^ In mice, it has been shown that this is likely due to the release of colony-stimulating factor 1 from sensory neurons after injury.^51^ Rodent models suggest that this microglial activation contributes to neuropathic pain^50 51^; however, there is still significant uncertainty as to whether human microglia are similarly activated, with data from imaging,^52^ postmortem tissue^53^ and cerebrospinal fluid analyses^54^ remaining inconclusive.

### Lymphoid cells

T and natural killer (NK) cells have been reported to be involved in both the disease progression and resolution of neuropathic pain in mice, depending on their activation state. For example, resolution of chemotherapy-induced neuropathy was shown to crucially depend on CD8^+^ T cells,^55^ while depletion of regulatory T cells increased hypersensitivity in a traumatic nerve injury model.^56^ NK cells have been reported to both exacerbate pathology, through cytotoxic granule release and inflammatory mediator production,^57^ as well as support recovery, through clearing of damaged neuronal debris and aberrant immune cells.^58^ Evidence from human studies is much more limited: T cells are over-represented in both plantar human nerves in focal neuropathy and wrist subsynovial connective tissue in carpal tunnel syndrome.^48 59^ However, the precise subtype of these cells and whether their presence is positively or negatively correlated with neuropathic pain remains unclear.

In contrast, pathological B-cell products, in the form of autoantibodies, have been more thoroughly studied in human neuropathy cohorts. It is clear that autoantibodies are present in a variety of conditions, including Guillain-Barré syndrome^60^ and chronic inflammatory demyelinating polyradiculopathy.^61^ There is also evidence that these antibodies can be directly linked to pain, with studies reporting that plasma exchange can act as an analgesic.^62^ Generally, this is a very active area of research, with many new auto-antibodies and mechanisms being reported over the past decade.^62^ For instance, a recent preprint identified that individuals with painful small fibre neuropathy and antibodies against fibroblast growth factor receptor 3 were more likely to show higher rates of abnormal sural nerve conduction than a seronegative small fibre neuropathy control group.^63^

### Glial cells

Besides immune cells, many studies of neuropathic pain have also investigated the role of glial cells, specifically Schwann cells, which myelinate peripheral nerves, satellite glial cells, which wrap around peripheral neuron cell bodies in dorsal root ganglia, and their equivalent cell types in the central nervous system: oligodendrocytes and astrocytes. Schwann cells have been implicated in peripheral neuron regeneration since the 1960s^64^ and experimental neuropathic pain models since the early 2000s.^65^ Recent studies with human samples agree that Schwann cell numbers are altered in peripheral nerves^47 66^ and their skin innervation territories^67 68^ in a variety of neuropathies. However, a correlation with pain was either not measured^47 66^ or was not observed,^67 68^ suggesting that Schwann cell numbers in skin are not linked to pain at effects large enough to be detectable with n=20–30 participants. Similar to Schwann cells, most of the evidence linking satellite glial cells to neuropathic pain derives from animal models. It is clear that satellite glial cells alter their transcriptional signature in models of nerve injury,^69^ and their activation has been linked to neuropathic pain in rodents, although mostly using correlational data.^70^ Finally, central nervous system glial cells have mainly been studied in the context of central sensitisation, and pretty much exclusively in rodents. Thus, spinal astrocyte activation, termed ‘astrogliosis’, has been reported in traumatic nerve injury models since the 1990s,^71^ while alterations in oligodendrocyte numbers and spinal cord myelination have been observed in about a dozen rodent studies, according to a recent review with systematic search.^72^ However, in the absence of stronger causal evidence and/or correlational studies in humans, the involvement of these cell types in neuropathic pain states remains somewhat uncertain.

#### Stromal cells

In spite of early work,^38^ interest in stromal cells, like fibroblasts, mural cells and endothelial cells, has flagged for several decades^73^ until a recent revival. There are now a number of studies that implicate fibroblast-neuron interactions in the pathophysiology of painful conditions. For example, fibroblasts can increase the excitability of mouse and human sensory neurons,^74 75^ have been linked to pain in rheumatoid arthritis^76^ and neuropathic pain in animal models.^77 78^ Moreover, expansion of a perineurial fibroblast population can be observed in sural nerves of human donors with polyneuropathy.^47^ The same publication also demonstrates proliferation of mural cells in neuropathy,^47^ a finding that translates to an animal model of neuropathic pain.^79^ In line with this, conditioned medium of a human mural cell line activated by TNF can induce intracellular changes in human stem-cell-derived sensory neurons.^79^ Future work needs to elucidate the role of various different stromal cell subpopulations—and whether some are more ‘proalgesic’ than others. Moreover, it remains to be tested in clinical populations whether there is a link between stromal cell proliferation and the likelihood of living with a painful, rather than a painless neuropathy.

## Conclusions

Overall, just like inflammation biology and immunology, the neuroscience of pain has benefitted from conducting more tissue-specific studies that explore the involvement of the local environments in which sensory neurons are located. More work remains to be conducted in this area, with a particular need for larger-scale human cohorts that will permit the detection of moderate-to-small correlations between a specific cell or molecular phenotype and neuropathic pain intensity or characteristics. There have already been efforts to harmonise and publicise protocols in this area to facilitate such studies.^80^,^82^ This will also permit better powered studies of the impact of age and being of the female sex; both increase the risk of living with neuropathic pain,^83^ but the precise pathophysiological drivers for this are difficult to establish conclusively without the use of large sample sizes.

There is now much stronger evidence for the involvement of stromal cells, as well as for specific macrophage subpopulations, opening up avenues for biologic drug development. For instance, given that several pathological fibroblast subsets are universal,^84^ it is possible that drug development efforts targeted at stromal cells in other areas such as arthritis or cancer^85 86^ will ultimately benefit those living with neuropathic pain. Equally, compounds aimed at disrupting abnormal cross-talk between sensory neurons, fibroblasts or resident immune cells are likely to relieve pain not only in neuropathic conditions, but also in other inflammatory diseases in which pain can persist in the absence of classical inflammation, for example, rheumatoid arthritis.^87^

The search for ion channel targets directly aimed at reducing sensory neuron hyperactivity is also continuing. Suzetrigine, a sodium channel blocker recently approved for acute pain by the Food and Drug Administration, has unfortunately failed to show reductions in neuropathic pain compared with placebo in a phase II study of individuals with radiculopathy (n=100–102), although this study was reportedly not powered to detect between-group effects.^88^

Finally, just like with immune-mediated inflammatory diseases, it is likely that future neuropathic pain treatments would benefit significantly from a stratified approach. In an ideal scenario, we would identify molecular and/or imaging biomarkers of which cell (sub)types are most implicated in particular patient subgroups and treat them differentially. Concerted, cross-disciplinary collaborative approaches can help us reach this goal sooner rather than later.
